# The relationship among acute-phase response proteins, cytokines and hormones in cachectic patients with colon cancer

**DOI:** 10.1186/1477-7819-8-85

**Published:** 2010-09-28

**Authors:** Ozgur Kemik, Aziz Sumer, Ahu Sarbay Kemik, Ismail Hasirci, Sevim Purisa, Ahmet Cumhur Dulger, Baris Demiriz, Sefa Tuzun

**Affiliations:** 1 Department of General Surgery, Yuzuncu Yıl University Medical Faculty, Van, Turkey; 2 Department of Biochemistry, Cerrahpasa Medical Faculty, University of Istanbul, Istanbul, Turkey; 3 Department of Biostatistics, Istanbul Medical Faculty, University of Istanbul, Istanbul, Turkey; 4 Department of Gastroeneterology, Yuzuncu Yıl University Medical Faculty, Van, Turkey; 5 Genaral Surgery, Zekai Tahir Burak Women Healty Researh and Education Hospital, Ankara, Turkey; 6 II. General Surgery, Haseki Research and Education Hospital, Istanbul, Turkey

## Abstract

**Backgraund:**

Acute-phase response proteins (APRP), cytokines and hormones have been claimed to be an independent prognostic factor of malignancies, however the basis for their association with prognosis remains unexplained. We suggest that in colon malignancies, as similar to pancreatic and lung cancers, changes in APRP are associated with angiogenesis.

**Methods:**

C-reactive protein (CRP), albumin, IL-1α, IL-1β, IL-6, IL-8, IL-10, TNF-α, midkine, VEGF-A, VEGF-C, leptin, adiponectin, and ghrelin serum levels are studied in 126 colon cancer patients and 36 healthy subjects.

**Results:**

We found statistically significant difference and correlations between two groups. We found significantly higher serum CRP, IL-1α, IL-1β, IL-6, IL-8, IL-10, TNF-α, VEGF-A, VEGF-C and leptin concentrations in patients relative to controls (p < 0.001). We found lower levels of the serum albumin, midkine, adiponectin and ghrelin in patients compared to control subjects (p < 0.001).

**Conclusions:**

Cachexia in patients with colon cancers is associated with changes in APRP, cytokines and hormone concentrations. These biomarkers and cachexia together have a direct relationship with accelerated angiogenesis. This may lead to a connection between the outcomes in malignancies and the biomarkers.

## Introduction

Cachexia due to cancer is one of the most frequent features of malignancy [1], it accounts up to 30-50% of cancer-related deaths in gastrointestinal tract malignancies [2]. Cachexia due to cancer is a complex metabolic disorder, including loss of adipose tissue due to lipolysis, loss of skeletal muscle mass, elevation of resting energy consumption, anorexia, and reduction of oral food intake [3,4].

Despite intensive studies that have been conducted thus far in this field, the multi-factorial pathological mechanism of cancer-related cachexia has not been fully exhibited, besides currently available treatment modalities remain profoundly unsatisfactory [5]. Nevertheless, it is well known that cytokine up-regulation contributes to involuntary weight loss, which is a hallmark of cancer-related cachexia [6,7]. Although the catabolism is mainly mediated by the effects of certain cytokines, such as tumor necrosis factor-α (TNF-α), interleukin-1β (IL-1β), and interleukin-6 (IL-6) [4,8], the mechanisms associated with cancer related anorexia are still not elucidated completely [9]. Previous studies concerning cachexia in gastrointestinal cancer revealed that other pro-inflammatory cytokines, such as IL-8 and, probably, vascular endothelial growth factor-A (VEGF-A) and midkine, might be involved in the process of cachexia [10]. Also, the proteins such as cytokines, some hormones and neuropeptides, which affect various central mechanisms, are tightly related to the regulation of the energy homeostasis [11]. These hormones include adiponectin, ghrelin, and leptin [11,12].

Adiponectin is a member of a group of proteins secreted from adipocytes [13] and its serum levels are (5-30 μg/ml) higher in women compared with men [14-16]. Adiponectin serum levels inversely correlate to body weight. Thus, low adiponectin levels are found in obesity [14,15], and high levels are found in anorexia nervosa [16] and during weight loss [17]. Several reports have indicated association between low adiponectin levels and increased risk of breast, endometrium, and gastric cancers [18-20]. The mechanisms responsible for regulation of adiponectin levels have not been fully elucidated. Yet, recent data suggest down-regulation of adiponectin by TNF-α, as well as by insulin [21]. The hormone ghrelin is a 28 amino-acid peptide, unique by the esterification of its third serine residue by n-octanoic acid [22]. The major source of ghrelin is the stomach, where it is synthesized in identical endocrine cells. The peptide is a potent inducer of growth hormone (GH) release, acting at the pituitary and hypothalamic levels [23].

Ghrelin participates directly in hypothalamic regulation of nutrition, it causes weight gain by reducing food utilization, increasing food intake, and inhibiting leptin-induced feeding reduction [24]. High ghrelin levels are associated with various cachectic states, such as anorexia nervosa and severe congestive heart failure [25]. Elevated levels were recently reported in lung cancer-induced cachexia [26], and in a cohort of male patients with mainly lung and prostate cancer [27].

Leptin is another member of the adipo-cytokines family. It is produced mainly by differentiated adipocytes and acts in the central nervous system as a suppresser for food intake and stimulator of energy consumption [12]. Leptin plasma levels are reported to be higher in anorexia nervosa patients [28], but lower in gastrointestinal [29], and pancreatic cancer patients [30]. Association between acute-phase response proteins (APRPs) and weight loss in cancer-related cachexia has been reported only in pancreatic carcinoma and melanoma [31].

The aim of our study was to evaluate associations between cachexia due to weight loss and APRPs (albumin, C-reactive protein (CRP)), cytokines (IL-1α, IL-1β, IL-6, IL-10, TNF-α, IL-8, VEGF-A, VEGF-C), midkine and hormones (adiponectin, leptin and ghrelin) in a population of newly diagnosed colon cancer patients.

## Materials and methods

### Patients

A total of 126 patients with colon cancer were enrolled in our study. Exclusion criterias included: previous treatment with chemotherapy, radiotherapy, or a major operation history 6 months before recovery; brain metastasis; second malignancy; acute or chronic infections; dysphagia; other primary cachectic states (i.e. congestive pulmonary disease, cirrhosis); elevated bilirubin or liver enzymes (> 2 of the upper normal reference value); renal failure (creatinine > 2 mg/dl); history of eating disorders; or gastrectomy.

Demographic clinical and anthropometric data were collected during recovery period. All pathology reports were reviewed, and data of the tumor histology were recorded. Stage was defined according to the 1997 American Joint Committee on Cancer Staging System [32].

We examined 41 cases of stage II cancers, 48 cases of stage III, 37 cases of stage IV cancers. There were 54 females and 72 males, with a median age of 56 years (range 38-74 years). We used sera from blood donors considered healthy on the basis of routine blood tests to obtain reference values in this study. The reference group consisted of 38 individuals, 16 females and 22 males, with a median age of 41 years (range 37-71 years).

Body mass index (BMI) was calculated as weight (kg) divided by height (m^2^), and cachexia was defined as ≥ 5% reduction in BMI at the time of recovery. The study protocol was approved by the medical Ethics Committee of Haseki Education and Research Hospital, Istanbul, and was in accordance with the ethical standards formulated in the Helsinki Declaration of 1975. Informed consent was obtained from all subjects.

### Analytical Methods

The concentrations of all parameters in the examined samples were measured in sera obtained from blood drawn in the fasting state, clotted (15 min, room temperature) and centrifuged (15 min, 1000 g). The serum samples were then immediately frozen at -80°C until further analysis (except albumin, CRP, and midkine).

The albumin concentrations were measured colorimetrically as a complex of albumin with bromocresol blue dye under acidic conditions. High-sensitive CRP was determined by the immunonephelometry (Behring Nephelometer II). Serum midkine concentrations were assayed with indirect ELISA R&D Systems, USA) anti-human midkine polyclonal antibodies were used. The serum concentrations of IL-1α, IL-1β, IL-6, IL-8, and TNF-α were assayed using a validated commercial ELISA (Quantikine R&D Systems, Mineapolis). The IL-10 levels were measured by using the Endogen Inc. assay (Cambridge). The concentrations of VEGF-A and VEGF-C were measured in duplicate with a commercially available quantitative sandwich enzyme immunassay kit (R&D Systems, USA). Adiponectin, ghrelin and leptin concentrations were determined using radioimmunassay kits (Linco Research, St. Charles, Missouri).

### Statistical Analysis

Data are presented as means ± SD. Comparisons were performed with the non-parametric Mann-Whitney *U*-test for continuous variables and with the χ^2 ^test for categorical data. Differences between groups were determined using the log-rank test. Two-sided p values < 0.05 were considered statistically significant.

## Results

There were no differences of median age between patients with cancer and subjects in control group (p > 0.05). None of the parameters showed significant difference when they were compared by age between those groups (p > 0.05). Plasma leptin levels showed no significant difference between genders (p > 0.05).

We found significantly higher serum CRP, IL-1α, IL-1β, IL-6, IL-8, IL-10, TNF-α, VEGF-A, VEGF-C and leptin concentrations in patients relative to controls (p < 0.001). We found lower levels of the serum albumin, midkine, adiponectin and ghrelin in patients compared to control subjects (p < 0.001).

We found favourable correlation between BMI loss and adiponectin levels (p < 0.01, r = 0.74). Also, we found positive correlation between midkine and albumin; similarly between both BMI loss and plasma leptin levels; and BMI loss and midkine.

There was significantly positive correlation between BMI loss and VEGF-A; as well as VEGF-A and IL-1.

VEGF-A and IL-6 correlation was similarly statistically significant; we also found favourable correlation between adiponectin and BMI loss.

(respectively; p < 0.01, r = 0.58; p < 0.001, r = 0.69, p < 0.01, r = 0.69; p < 0.01, r = 0.71; p < 0.01, r = 0.65, p < 0.001, r = 0.73; p < 0.01, r = 0.61). The Concentrations of all parameters in patients and controls were shown in table 1.

**Table 1:** Concentrations of all parameters in patients and controls

Parametres	Patients	Controls	p
Age (y)	43.5 ± 10.7	40.4 ± 11.3	> 0.05
Gender (F/M)	73/53	20/16	
CRP (ng/ml)	9.8 ± 4.3	3.5 ± 2.1	< 0.001
Albumin (g/dl)	2.5 ± 1.2	4.4 ± 1.1	< 0.001
Midkine (ng/ml)	0.21 ± 0.034	0.36 ± 0.1	< 0.05
VEGF-A (pg/ml)	629.3 ± 205.6	309.4 ± 135.8	< 0.001
VEGF-C (pg/ml)	3428.1 ± 987.5	1736.9 ± 685.8	< 0.001
IL-1α (pg/ml)	785.7 ± 243.9	209.6 ± 102.3	< 0.001
IL-1β (pg/ml)	693.9 ± 305.7	276.9 ± 132.2	< 0.001
IL-6 (pg/ml)	109.6 ± 45.8	34.9 ± 29.7	< 0.0001
IL-8 (pg/ml)	78.6 ± 25.4	29.5 ± 19.6	< 0.001
IL-10 (pg/ml)	5.7 ± 1.9	2.3 ± 1.1	< 0.001
TNF-α (pg/ml)	28 ± 14	10 ± 7	< 0.001
Leptin (ng/ml)	47.6 ± 10.3	23.8 ± 11.4	< 0.001
Adiponectin (μg/ml)	4.3 ± 2.5	6.5 ± 1.4	< 0.001
Ghrelin (pmol/l)	178.5 ± 89.6	300.9 ± 57.3	< 0.001

## Discussion

In our study, we analyzed the associations between acute-phase response cytokines, pro-inflammatory cytokines, cytokines, hormones and cancer related cachexia in a population of newly diagnosed or newly recurrent, untreated colon cancer. Systemic inflammation is a non specific process of many cancer types. Association between acute-phase related proteins and accelerated weight loss has been described only in a few cancer types; which are pancreatic, lung cancers and melanoma [31]. Decreased albumin concentrations are involved with cachexia and are a common laboratuary feature in gastrointestinal cancers. Hypoalbuminemia has recently been demonstrated to be a predictive factor of poor responsiveness [33,34]. Our results showing a weight-loss dependent association with cachexia may support the association of hypoalbuminemia and cachexia. The decrease in transferring concentrations seems to be especially weight-loss dependent. The ongoing systemic imflammatory response determined in terms of CRP concentrations has recently gained some interest, as an-easy-to measure and well-standardized outcome predictor [35,36]. Similar to substantial weight loss [10], an elevation in CRP concentration has been related to increased extent of primary tumor and has been associated with poor survival [35,36]. Our results may support the association of CRP concentrations and cancer related cachexia. The association of CRP with the pro-angiogenic environment may contribute to adverse effects together with CRP elevation [37]. Our results reveal a positive correlation between CRP and circulating IL-8, midkine, which both have pro-angiogenic properties [38]. Similarly CRP and VEGF correlation has been determined and these results may further support this hypothesis. It is also of interest that similar to CRP [35,36], circulating midkine [10] and IL-8 [39] have been found to reflect lymph node involvement in esophageal squamous cell carcinoma. The concentrations of circulating IL-6 and midkine, independently of the patients weight status, and with Il-1, Il-8 and VEGF in cachectic cancer patients. TNF-α, IL-1 and IL-6 are key cytokines involved in cancer-related cachexia. However, apart from IL-6, alterations in their systemic levels are rarely detected [31]. As experimental cytokine-directed anti-cachectic therapies yielded moderate results [31], there is a need for finding other mediators of cancer cachexia [5,31]. We found midkine and VEGF to be independent predictors of weight loss in patients with colon cancer. Our results provide evidence for an association of midkine and VEGF with systemic inflammation and malnutrition, supporting a possible involvement of these cytokines in the pathogenesis of cachexia. However, only the concentrations of VEGF, and leptin but not midkine, are associated with weight loss in the examined cohort of cancer patients. Midkine was related to inflammation and was correlated with albumin concentration, while the associations were not affected by cachexia. These together may further indicate VEGF as rather a pro-cachectic cytokine, corroborating the findings of other authors demonstrating VEGF associations with standard pro-cachectic cytokines. IL-1 and IL-6 have been implicated in the regulation of VEGF expression [40], while anti-TNF α treatment (infliximab) has been shown to decrease serum VEGF concentrations [41]. We found that VEGF correlated with IL-1 and IL-6 exclusively in cachectic colon cancer patiens. Although the involvement of midkine in inflammation is well-documented [42], only a weak correlation between midkine and CRP in cancer patients has been reported [43].

Adiponectin levels are reported to be inversely correlated with body weight. Thus, voluntary weight loss, as well as anorexia nervosa, is associated with elevated adiponectin levels [14,17,44]. However, in our study, we found no correlation between decreased BMI and adiponectin levels. Adiponectin levels are regulated mainly by changes in the adipose tissue [44]. The lack of association between adiponectin levels and weight loss may simply reflect the preservation of adipose tissue. Recent studies, which found inhibition of adiponectin secretion from adipocytes by various cytokines, including TNF-α, support our observations [21,45]. Thus, the lack of elevation of adiponectin levels after cancer related cachexia, may reflect altered regulation of adiponectin in this condition. Interestingly, lower adiponectin levels were also found in a cohort of cachectic patients with very advanced stage of lung cancer compared with healthy volunteers [46].

Elevated levels of total or active ghrelin in cancer related cachexia have been reported in cohorts of mainly male lung cancer patients [26].

In our study, we report elevated ghrelin levels in a cohort of colon cancer patients. Notably, high levels of ghrelin were also found among a significant number of cachectic lung cancer patients [26]. Our results suggest that measurement of ghrelin levels may have important clinical implications in treating cancer related cachexia syndrome. Although leptin levels are directly associated with weight loss after fasting [47], associations between leptin levels and cancer related cachexia are not yet fully elucidated. Thus, lower leptin levels were found in patients with gastrointestinal cancers, regardless of the degree of weight loss [48]. However, association between leptin levels and weight loss was noted in a cohort of lung [49], and pancreatic cancer patients [29]. We also found a correlation between leptin levels and cancer-induced weight loss. Our results showing a weight-loss dependent association with cachexia may support the association of leptin with cachexia. Moreover, leptin levels have positive correlation with IL-6 levels and CRP in our study. This situation may explain high IL-6 levels related with cancer progression and invasion. Pyrogenic activity of this proinflammatory cytokine may responsible for cachexia and weight loss. In addition, IL-6 stimulates to the synthesis of APRP. So, adiponectin, ghrelin and leptin levels accelerated by APRP levels. Association with between weight loss and levels of pro-inflammatory cytokines, cytokins, APRP, adiponectin, ghrelin and leptin leves have not been explained yet. We suggested that adiponectin, ghrelin and leptin are tightly regulated the energy homeostasis such as cytokines, which affect various central mechanisms [11]. Our study revealed an association between cytokine and pro-inflammatory cell concentrations and APRP in patients with colon cancer. In our study, there is an association between these parameters and levels of these hormones, which confirm our hypothesis.

In conclusion, our results provide evidence for an association between colon cancer related cachexia and changes in the concentrations of APRPs, cytokines and hormones. More studies should be performed to confirm this association between cachexia and APRP, cytokines, and hormones in patients with colon cancer, as well as in other cancer types.

## Competing interests

The authors declare that they have no competing interests.

## Authors' contributions

**OK, ST**- Collected data and wrote the manuscript in draft. **ASK**- Carried out the biochemical analysis. **SP**-Carried out the statistical analysis. **AS, ACD, IH and BD**- Took part in and contributed the discussion. All authors have read and approve of the final manuscript.
